# Effect of intraperitoneal infusion of ropivacaine combined with dexmedetomidine in patients undergoing total laparoscopic hysterectomy: a single-center randomized double-blinded controlled trial

**DOI:** 10.1007/s00404-023-07020-w

**Published:** 2023-04-01

**Authors:** Liyan Miao, Qiuchun Chen, Yuping Wang, Denggui Wang, Min Zhou

**Affiliations:** 1 Department of Anesthesiology, Fujian Maternity and Child Health Hospital College of Clinical Medicine for Obstetrics & Gynecology and Pediatrics, Fujian Medical University, 18 Daoshan Road, Gulou District, Fuzhou, Fujian China; 2 Department of Anesthesiology, Fujian Medical University Union Hospital, Fuzhou, China

**Keywords:** Ropivacaine, Dexmedetomidine, Intraperitoneal spray, Total hysterectomy, Quality of postoperative recovery

## Abstract

**Purpose:**

To investigate the effect of intraperitoneal infusion of ropivacaine combined with dexmedetomidine and ropivacaine alone on the quality of postoperative recovery of patients undergoing total laparoscopic hysterectomy (TLH).

**Methods:**

Female patients scheduled to undergo a TLH under general anesthesia at Fujian Maternity and Child Health Hospital were included. Before the end of pneumoperitoneum, patients were laparoscopically administered an intraperitoneal infusion of 0.25% ropivacaine 40 ml (R group) or 0.25% ropivacaine combined with 1 µg/kg dexmedetomidine 40 ml (RD group). The primary outcome was QoR-40, which was assessed before surgery and 24 h after surgery. Secondary outcomes included postoperative NRS scores, postoperative anesthetic dosage, the time to ambulation, urinary catheter removal, and anal exhaust. The incidence of dizziness, nausea, and vomiting was also analyzed.

**Results:**

A total of 109 women were recruited. The RD group had higher QoR scores than the R group at 24 h after surgery (*p* < 0.05). Compared with the R group, NRS scores in the RD group decreased at 2, 6, 12, and 24 h after surgery (all *p* < 0.05). In the RD group, the time to the first dosage of postoperative opioid was longer and the cumulative and effective times of PCA compression were less than those in the R group (all *p* < 0.05). Simultaneously, the time to ambulation (*p* = 0.033), anal exhaust (*p* = 0.002), and urethral catheter removal (*p* = 0.018) was shortened in the RD group. The RD group had a lower incidence of dizziness, nausea, and vomiting (*p* < 0.05).

**Conclusion:**

Intraperitoneal infusion of ropivacaine combined with dexmedetomidine improved the quality of recovery in patients undergoing TLH.

**Trial registration:**

ChiCTR2000033209, Registration Date: May 24, 2020.

## What does this study add to the clinical work

**Table:** 

Intraperitoneal spraying of ropivacaine combined with dexmedetomidine can improve the quality of recovery, postoperative analgesia, and accelerate discharge after general anesthesia for laparoscopic hysterectomy.	

## Introduction

Total laparoscopic hysterectomy (TLH) is widely used in the clinic, mainly due to its advantages, such as low trauma and postoperative complications, early ambulation, quick recovery, and short hospital stay. However, this is still an invasive treatment. Abdominal incision, intraoperative traction of the peritoneum and diaphragm, ischemia of internal organs, and hypoxia caused by increased intra-abdominal pressure, as well as traumatic, inflammatory reactions in the body can lead to acute postoperative pain [1]. Postoperative pain can cause psychological anxiety, affect comfort, and produce a series of pathological reactions, eventually leading to postoperative complications [2]. Pain after laparoscopic surgery is related to changes in abdominal wall tension, peritoneal traction, and local injury, and it includes incision trauma pain, inflammatory pain, neuropathic pain, etc. [3]. Therefore, based on the above characteristics of pain after laparoscopic hysterectomy, providing patients with safe and efficient postoperative analgesia is particularly critical.

Opioids have significant analgesic effects; however, they are dose-dependent, and it is impossible to ensure stable blood concentration. Extensive use of opioids after surgery may cause unnecessary side effects such as respiratory depression, excessive sedation, urinary retention, nausea, vomiting, dizziness, etc. [4]. Currently, reduced use of opioids is recommended to shorten the length of hospitalization and promote rapid recovery.

Clinically, spraying local anesthetics intraperitoneally under laparoscopy is considered an effective method for reducing postoperative pain [5, 6]. The main advantage of this approach is the nonexistence of adverse effects that commonly follow systemically administered opioids. Ropivacaine is a long-acting amide local anesthetic that can reduce postoperative visceral pain by blocking the splanchnic nerve from transmitting pain signals to the central nervous system. Relevant studies have shown [7, 8] that ropivacaine is safe and effective for local incision infiltration and abdominal spraying after laparoscopic surgery. However, as local anesthetics are short-acting, adjuvants are added to prolong the duration of anesthesia and enhance the analgesic effect.

Dexmedetomidine is an α_2_ adrenergic receptor agonist with sedative, analgesic, anti-anxiety, and anti-sympathetic nerve effects, and does not cause respiratory depression ^[9]^. Studies have shown that topical application of dexmedetomidine during nerve block can enhance the analgesic effect of local anesthetics, reduce the dosage of local anesthetics and improve the postoperative analgesic effect [10]. In addition, dexmedetomidine combined with ropivacaine for peripheral nerve block can prolong the action of local anesthetics without neurotoxicity [11].

While intraperitoneal local anesthetics (IPLA) have been widely accepted and used, no previous study evaluated the effects of adjuvants in this form of anesthesia. Based on the above studies, we hypothesized that intraperitoneal spraying of ropivacaine combined with dexmedetomidine could be a new technique for analgesia after laparoscopic hysterectomy. Therefore, this study aimed to analyze the effect of intraperitoneal infusion of ropivacaine combined with dexmedetomidine on the quality of postoperative recovery of patients undergoing TLH.

## Methods

### Study design

Ethics Committee of Fujian Maternity and Child Health Hospital College of Clinical Medicine for Obstetrics & Gynecology and Pediatrics, Fujian Medical University approved this study (Ref. 2020YJ164). Each participant provided informed consent.

Women who met the ASA I–II, aged 18–65 years, scheduled for elective laparoscopic hysterectomy for uterine fibroids under spinal anesthesia were enrolled. This single-center trial was conducted at Fujian Maternity and Child Health Hospital between June 2020 and April 2021. Exclusion criteria were allergy to ropivacaine, dexmedetomidine, or other anesthetics; patients with hypertension, diabetes, heart disease, or chronic pain; mental disorders and communication disorders; conversion to open surgery; severe renal and/or hepatic disease.

### Randomization and masking

This was a single-center prospective, double-blind, randomized controlled trial. Patients were randomized using a computerized simple randomization scheme in a 1:1 ratio to the R and RD groups. The group sequence was concealed in sealed opaque envelopes, which were opened only after obtaining informed consent. They received either 40 ml 0.25% ropivacaine (R group) or 40 ml 0.25% ropivacaine and 1 µg/kg dexmedetomidine mixture (RD group) [12, 13]. The study drug was prepared by an anesthesiologist who was not involved in the study. The patient, attending anesthesiologists, surgeons, recovery ward nurses, data collectors, and the person who performed the final statistical analysis were all blinded to group assignment.

### Anesthesia and surgical techniques

The two groups of patients routinely abstained from drinking and fasted before the operation. Noninvasive blood pressure, pulse frequency, peripheral oxygen saturation, end-tidal gas monitoring, electrocardiography, and the bispectral index (BIS) were monitored throughout the procedure. One peripheral iv-lines was inserted, and 200–300 ml sodium lactate Ringer’s solution was injected. Endotracheal intubation was performed after the injection of midazolam 2 mg, sufentanil 0.5 µg/kg, etomidate 0.3 mg/kg, and rocuronium 0.6 mg/kg. After intubation, the anesthesia machine was used for mechanical ventilation, VT 6–8 ml/kg, RR 10–12 times/min, I:E 1:2, oxygen flow rate 1 L/min, and P_ET_CO_2_ maintained at 35–45 mmHg. During the operation, intravenous anesthesia was maintained, and propofol 4–8 mg kg^−1^ h^−1^ and remifentanil 0.1–0.25 μg kg^−1^ min^−1^ were continuously pumped according to the change of MAP and HR of the patients. The BIS was maintained at 40–60. Rocuronium 10 mg was intermittently injected during the operation. Moreover, 6 mg of ephedrine was given when MAP < 60 mmHg, and 0.5 mg of atropine was given when HR < 50 times/time.

### Intervention

Intra-abdominal hemostasis was completed before the end of the operation, during which the R group was sprayed with 0.25% ropivacaine 40 ml, and the RD group was sprayed with 0.25% ropivacaine and 1 µg/kg dexmedetomidine mixture 40 ml under laparoscopy. The surgeon sprayed drugs through an epidural catheter, aiming to cover the whole peritoneum with the liquid. After the operation, the tracheal tube was removed after the patient resumed spontaneous breathing. Thereafter, patients were connected to a patient-controlled analgesia pump with 1 μg/kg sufentanil 0.9% normal saline (100 ml of total volume) to deliver a bolus of 5 ml above the analgesics, with continuous background infusion of 2.5 mL/h, and a lockout time of 15 min. All patients were encouraged to use this pump so that the pain was mild. The same surgeon with more than 15 years of work experience performed all operations. The nurses recorded all the signs and symptoms of systemic toxicity induced by local anesthetics.

### Outcomes and measurement

The primary outcome was the quality of recovery, which was assessed on the day before surgery and 24 h after surgery using a 40-item questionnaire (QoR-40) [14]. The total score ranged from a minimum of 40 to 200 points, respectively, representing the worst and best recovery quality.

Secondary outcomes were postoperative NRS score, the time to the first dosage of postoperative opioid, and the cumulative number and the effective number of PCA compressions 24 h after the operation. NRS score is a numeric rating scale patients use to evaluate their pain on a scale from 0 to 10, with 0 indicating no pain and 10 indicating the worst imaginable pain. The time to ambulation, anal exhaust, and urethral catheter removal were also evaluated. The incidence of dizziness, nausea, and vomiting was also analyzed.

### Statistical analysis

Our sample size calculation for the two-tailed testing was based on the global QoR-40 score. A 10-point difference represents a clinically relevant improvement in the quality of recovery based on data from a previous study [14]. A mean (standard deviation) of the QoR score at 24 h postoperative equivalent to 171 (14.6) was estimated based on our pilot study. A power analysis with a type I error estimate of 5% (alpha = 0.05), and a power (1-Beta) of 90% indicated that a sample of 46 subjects per group was required. Allowing for approximately 10% incomplete follow-up or dropout, 112 subjects were enrolled in this study.

SPSS version 18.0 was used to perform statistical analysis (SPSS Inc., Chicago, IL, USA). The normality of distribution was assessed with the Kolmogorov–Smirnov test. Parameters with normal distribution were reported as mean ± standard deviation (SD) and analyzed with the independent test, and those with non-normal distribution were reported as the median and interquartile range (IQR) and analyzed using the Mann–Whitney test. Categorical variables were reported as the number of patients (%) and evaluated using the *X*^2^ or Fisher’s exact test when appropriate. A *p* < 0.05 was considered statistically significant.

## Results

**Fig. 1:**
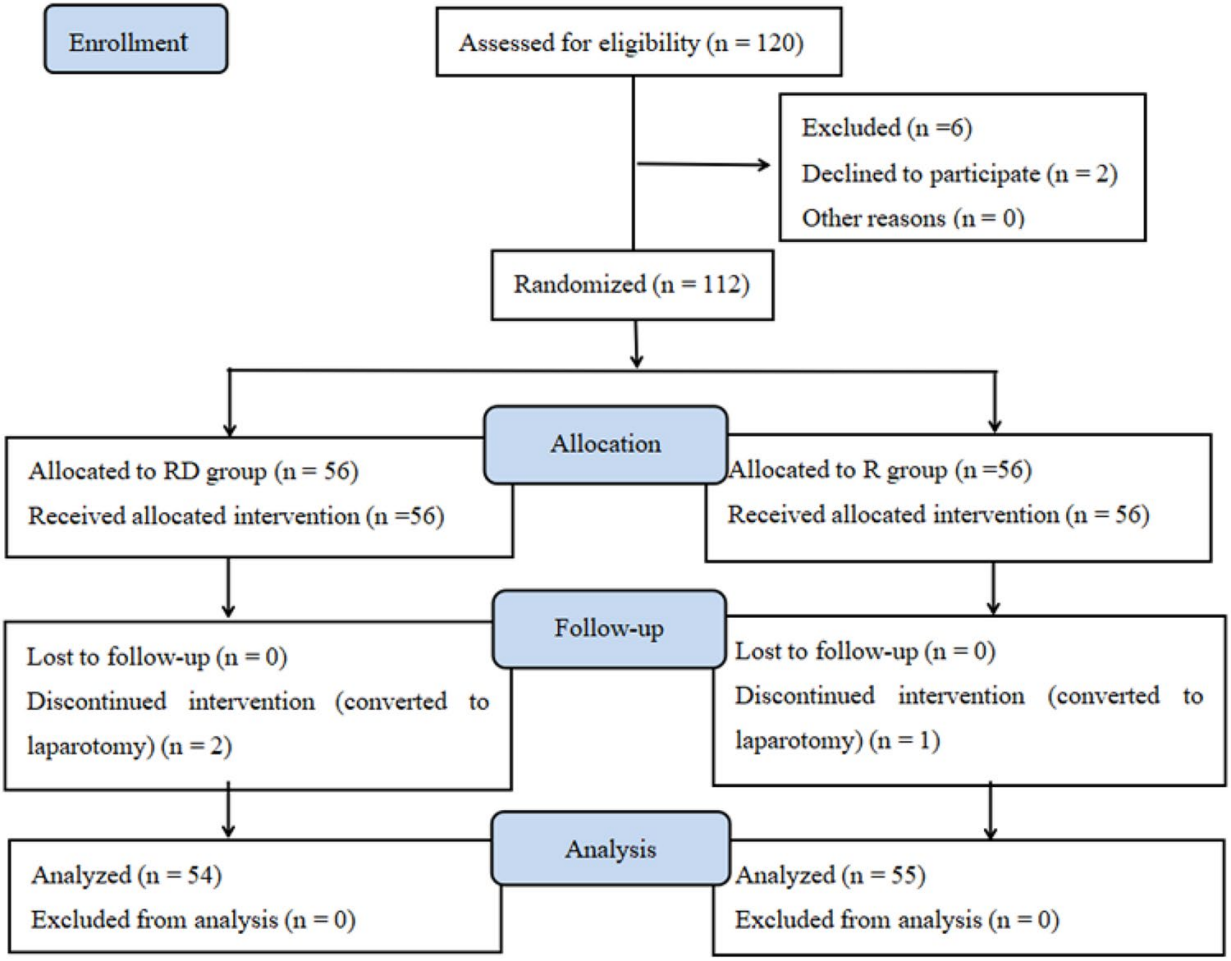
Flow diagram depicting the progress of the subject through the trial

Among 120 patients who were initially assessed for eligibility to participate in the study, 6 patients did not meet the inclusion criteria because of hypertension and diabetes, 2 declined to participate, and the remaining 112 patients were enrolled in the study. In addition, two patients from the RD group and one patient from the R group were later excluded because of intraoperative conversion to laparotomy. Eventually, 109 patients were included in the final analysis (see Fig. 1). The two groups had no significant difference in the baseline data (all *p* > 0.05, Table 1).

**Table 1:** Patient characteristics and surgery details

	RD group (*n* = 54)	R group (*n* = 55)	*p *value
Age (year)	51.7 ± 3.9	51.4 ± 5.0	0.708
ASA (I/II)	52/2	53/2	1.0
BMI (kg/m^2^)	29.1 ± 1.9	28.9 ± 2.0	0.652
Preoperative QoR-40	182.7 ± 4.8	180.8 ± 5.1	0.060
Duration of surgery (min)	105.7 ± 5.5	107.4 ± 5.1	0.086
Duration of anesthesia (min)	116.4 ± 4.7	114.9 ± 4.9	0.096

Patients in the RD group had significantly higher QoR scores at 24 h after surgery (*p* < 0.05). As shown in Table 2, the QoR-40 scores [mean (SD)] were 173.4 (9.1) and 164.1 (10.6) in the RD group and the R group, respectively. The improvement in QoR scores in the RD group reflected improvements in emotional status, physical comfort, psychological support, and postoperative pain. However, the two groups had no significant differences in physical independence dimensions (Table 2).

**Table 2:** Comparison of 24 h QoR-40 scores between the two groups

	RD group (*n* = 54)	R group (*n* = 55)	*p *value
Emotional status	43.4 ± 4.9	40.7 ± 5.0	0.005
Physical comfort	52.1 ± 6.3	48.0 ± 7.5	0.003
Psychological support	31.6 ± 3.0	30.0 ± 3.8	0.017
Physical independence	16.2 ± 1.9	16.8 ± 1.6	0.099
Pain	30.3 ± 2.2	28.9 ± 1.8	0.001
Total	173.4 ± 9.1	164.1 ± 10.6	< 0.001

Postoperative pain scores in the RD group decreased at 2, 6, 12, and 24 h after surgery compared with the R group (*p* < 0.05). In addition, in the RD group, the time to the first dosage of postoperative opioid was longer, while the cumulative and effective times of PCA compression were shorter than those in the R group (all *p* < 0.05) (Table 3).

**Table 3:** Comparison of postoperative pain between the two groups

	RD group (*n* = 54)	R group (*n* = 55)	*p *value
NRS			
2 h	1(1,2)	2(2,2)	< 0.001
6 h	3(2,4)	5(3,6)	< 0.001
12 h	2(2,4)	4(3,5)	< 0.001
24 h	1(1,2)	2(2,4)	< 0.001
The time to first dosage of postoperative opioid (h)	4.5 ± 0.5	4.1 ± 0.6	< 0.001
Cumulative times of PCA compression (time)	10.3 ± 1.9	11.1 ± 2.0	0.033
Effective times of PCA compression (time)	7.9 ± 1.3	8.6 ± 1.9	0.029

Compared with the R group, the time to ambulation, anal exhaust, and urethral catheter removal were shortened, and the incidences of dizziness, nausea, and vomiting were decreased in the RD group (*p* < 0.05) (Table 4).

**Table 4:** Comparison of hospitalizations between the two groups

	RD group (*n* = 54)	R group (in = 55)	*p *value
Time to ambulation (h)	21.5 ± 1.5	22.1 ± 1.4	0.033
Time to anal exhaust (h)	24.0 ± 1.7	25.0 ± 1.5	0.002
Time to urethral catheter removal (h)	21.9 ± 1.4	22.6 ± 1.2	0.018
Dizziness	6 (11.1%)	18 (32.7%)	< 0.001
Nausea and vomiting	5 (9.3%)	14 (25.5%)	< 0.001

In addition, no patient in either group had any signs or symptoms of ropivacaine toxicity. No significant adverse reactions were associated with dexmedetomidine, such as bradycardia, hypotension, and temporary hypertension.

## Discussion

Our results showed that intraperitoneal application of ropivacaine and dexmedetomidine could significantly enhance the QoR-40 and postoperative analgesia in patients undergoing TLH. In addition, the combination of ropivacaine and dexmedetomidine reduced the incidence of general anesthesia-induced side effects, such as dizziness, nausea, and vomiting, and shortened the duration of the recovery of intestinal and urinary function.

The main point of the QoR-40 scale is to evaluate the postoperative recovery quality of patients. It is used to measure the recovery of patients after anesthesia from five aspects, i.e., emotional state, physical comfort, psychological support, and self-care ability, and pain. The measure is suitable for different genders, ages, and cultural backgrounds, and its effectiveness has been clinically verified [15, 16]. QoR-40 is the most comprehensive assessment scale for evaluating a patient’s overall health status postoperatively, where higher scores indicate better post-surgical recovery ^[15]^. This study showed statistically significant differences in QoR-40 scores at 24 h after surgery between the RD and R group, suggesting that intraperitoneal spraying with ropivacaine and dexmedetomidine could relieve postoperative pain, improve postoperative emotional state and physical comfort, and improve postoperative recovery quality. These findings support using ropivacaine and dexmedetomidine for early postoperative pain management and postoperative rehabilitation.

If postoperative pain is not timely and effectively treated, the hospital stay will be prolonged, which is not conducive to the rapid recovery of patients. Previous studies have confirmed that intraperitoneal anesthesia with ropivacaine can significantly reduce postoperative pain after laparoscopic cholecystectomy [17, 18]. Other studies reported that intraperitoneal spraying and incision infiltration with ropivacaine could significantly reduce postoperative pain in patients with cervical cancer by blocking the visceral nerve conduction pathway, reducing the neuroendocrine response after surgical stimulation and the release of pain mediators [19]. A review of intraperitoneal instillation of local anesthetics after laparoscopic gynecologic surgery reported an overall reduction in pain scores at 1 to 2 h, with no difference observed at 24 h postoperatively, indicating that local anesthetics merely have a temporary effect [20].

The results of the present study revealed that an intraperitoneal spray of ropivacaine combined with dexmedetomidine could more effectively reduce the pain within 24 h after the operation, prolong the time of the first postoperative additional analgesia, and reduce the dosage of postoperative analgesics compared with an intraperitoneal spray of ropivacaine alone. The analgesic mechanism of topical dexmedetomidine is not fully clear. However, it may inhibit or reduce the release of substance P and norepinephrine, prevent the transduction of nociceptive pain signals, and inhibit the action potential of the nerve cell membrane by blocking the sensitive voltage-gated sodium channel of nerve endings on the inner surface of the pelvic cavity, thus enhancing the local anesthetic effect of ropivacaine [21]. In addition, the synergistic analgesic effect of dexmedetomidine may be because it causes local vasoconstriction, leading to the increase of local anesthetic concentration around the local nerve, thus enhancing the analgesic effect.

The present study found that the time to ambulation, anal exhaust, and urethral catheter removal shortened, and the incidence of dizziness, nausea, and vomiting decreased in the RD group. Poor pain control after laparoscopic surgery increases postoperative inflammatory stress response, leading to delayed recovery of gastrointestinal function and prolonged hospital stay. Postoperative nausea and vomiting affect the patients’ eating, wound healing, life quality, and satisfaction [22]. Intraperitoneal spraying of ropivacaine with dexmedetomidine may have an analgesic and anti-inflammatory role during the perioperative period by blocking the visceral nerve conduction pathway, reducing the neuroendocrine reaction after surgical stimulation, and accelerating the recovery of postoperative gastrointestinal function. In addition, dexmedetomidine can regulate the release of the neurotransmitter serotonin, which may be involved in alleviating postoperative nausea and vomiting [23].

There are some limitations to this trial that should be considered when interpreting the results. First, a single dose of dexmedetomidine was given in our study. Hence, more doses and long-term effects of dexmedetomidine should be examined. The type and dose of local anesthetic and dexmedetomidine used in our study were based on previous studies. However, there were no obvious adverse reactions at these doses, revealing them to be safe, just as the administration method. Second, this was a single-center study in a strictly defined patient population, which may potentially limit the external validity of the findings. Third, the specific location, mode, and time changes of pain after laparoscopic surgery were not recorded. This may be useful for guiding postoperative analgesia and will be further optimized in future studies.

## Conclusions

Intraperitoneal spraying of ropivacaine combined with dexmedetomidine can improve the quality of recovery, postoperative analgesia, and accelerate discharge after general anesthesia for laparoscopic hysterectomy. However, further studies are required to evaluate different doses and optional infusion time of dexmedetomidine gynecological laparoscopic surgery.

## Data Availability

All data generated or analyzed during this study are included in this published article.

## Abbreviations

ERAS: Enhanced recovery after surgery
SD: Standard deviation
IQR: Interquartile range
